# The Cardiovascular Response to Interval Exercise Is Modified by the Contraction Type and Training in Proportion to Metabolic Stress of Recruited Muscle Groups

**DOI:** 10.3390/s21010173

**Published:** 2020-12-29

**Authors:** Benedikt Gasser, Daniel Fitze, Martino Franchi, Annika Frei, David Niederseer, Christian M. Schmied, Silvio Catuogno, Walter Frey, Martin Flück

**Affiliations:** 1 Departement für Sport, Bewegung und Gesundheit—Abteilung Rehabilitative und Regenerative Sportmedizin—Universität Basel—Birsstrasse, 320B CH, 4052 Basel, Switzerland; benediktandreas.gasser@unibas.ch; 2 Laboratory for Muscle Plasticity, Departement of Orthopaedics Balgrist Campus, University of Zurich Lengghalde, 8008 Zürich, Switzerland; daniel.fitze@oym.ch (D.F.); annika.frei@ksw.ch (A.F.); silvio.catuogno@balgrist.ch (S.C.); walter.frey@balgrist.ch (W.F.); 3 Balgrist University Hospital Forchstrasse 319, 8008 Zürich, Switzerland; 4 Department of Biomedical Sciences, University of Padova, 35131 Padua, Italy; martino.franchi@unipd.it; 5 Sports Cardiology Section, Department of Cardiology, University Heart Center Zurich, University Hospital Zurich, University of Zurich, 8091 Zurich, Switzerland; david.niederseer@usz.ch (D.N.); christian.schmied@usz.ch (C.M.S.)

**Keywords:** eccentric, concentric, muscle contraction

## Abstract

Background: Conventional forms of endurance training based on shortening contractions improve aerobic capacity but elicit a detriment of muscle strength. We hypothesized that eccentric interval training, loading muscle during the lengthening phase of contraction, overcome this interference and potentially adverse cardiovascular reactions, enhancing both muscle metabolism and strength, in association with the stress experienced during exercise. Methods: Twelve healthy participants completed an eight-week program of work-matched progressive interval-type pedaling exercise on a soft robot under predominately concentric or eccentric load. Results: Eccentric interval training specifically enhanced the peak power of positive anaerobic contractions (+28%), mitigated the strain on muscle’s aerobic metabolism, and lowered hemodynamic stress during interval exercise, concomitant with a lowered contribution of positive work to the target output. Concentric training alone lowered blood glucose concentration during interval exercise and mitigated heart rate and blood lactate concentration during ramp exercise. Training-induced adjustments for lactate and positive peak power were independently correlated (*p* < 0.05, |r| > 0.7) with indices of metabolic and mechanical muscle stress during exercise. Discussion: Task-specific improvements in strength and muscle’s metabolic capacity were induced with eccentric interval exercise lowering cardiovascular risk factors, except for blood glucose concentration, possibly through altered neuromuscular coordination.

## 1. Introduction

In untrained subjects, repeated sessions of continuous muscle work produce a number of systemic reactions that enhance aerobic capacity [1,2]. For instance, typical improvements in oxygen uptake and aerobic power output in the order of 10–15% can be observed after few weeks of endurance training consisting of repeated sessions of continuous exercise at moderate intensity [1,2]. The thereby observable adaptations of systemic aerobic capacity are matched to larger increases in cardiac output and gains in skeletal muscle’s aerobic capacity [3]. 

The cardiovascular and cellular adaptations in repeatedly exercised muscle groups stand in a positive and negative relationship with the intensity and volume of the exercise performed during the workouts [4,5,6] and the initial level of fitness [4]. However, attainable metabolic improvements by endurance training often come at the detriment of muscle strength due to an interference effect [7,8]. Often the gains in endurance performance are limited by the inherent moderate fatigue resistance of untrained subjects that do not allow performing exercise at the high intensities or long durations that induce large degrees of adaptations [9]. 

Lengthening (i.e., eccentric) types of muscle contraction allow to specifically increase the net mechanical stimulus impacting on exercised skeletal muscle by a factor of up to four compared to ‘conventional’ shortening (i.e., concentric) types of exercise [10]. The effect is explained by a higher metabolic efficiency of force development during lengthening than shortening type muscle contractions [11]. This biomechanical characteristic permits performing a comparable total of positive and negative work at a lower cardiac output [12,13,14]. In consequence, resistance-training paradigms based on eccentric contractions allow specifically enhancing muscle strength compared to concentric training at a comparable total workload [13], offering a distinct advantage to ameliorate the capacity to prevent the risk of falls with gravity-related mechanical perturbations [15]. Importantly, endurance-training protocols based on continuous exercise under eccentric contractions also bear advantages compared to concentric contractions in promoting gains in muscle performance along with those of endurance-related traits with the training of unfit subjects [15]. The implicated processes include specific improvements of glucose handing rather than gains in systemic aerobic capacity [6].

However, the combination of eccentric contractions with an endurance stimulus also comes with certain disadvantages for the metabolic effects of training. For instance, due to the concomitantly lower metabolic load, eccentric type endurance training at moderate intensity may dampen, or even prevent, the typical increases in the volume density of mitochondria that can be observed with work-matched concentric endurance training [6,7,16]. This ‘maladaptation’ potentially interferes with the improvement in the avoidance of lactate accumulation at high exercise intensities. As well, the vaso-restrictive effect of eccentric contractions of large leg muscle groups [10,12] may increase systolic blood pressure during the ejection phase (i.e., afterload) of the cardiac cycle [2,17,18] and elevate the blood concentration of glucose during eccentric exercise in untrained subjects [19,20,21]. These acute reactions pose potential health risks to subjects with cardiac or metabolic limitations, potentially counter-indicating the application of eccentric training paradigms [22]. In this respect, it is striking that the amelioration of insulin sensitivity and the lowered blood glucose concentration with repeated sessions of eccentric resistance training is preceded by acute insulin resistance in untrained subjects after intense eccentric exercise [23,24]. It is unclear whether alterations in the cardiovasculature, such as compromised hemodynamic parameters and glucose handling, are inter-related to adjustments in metabolic functioning of recruited muscle with endurance types of eccentric exercise.

Interval-types of exercise are a potential venue to circumvent metabolic limitations of eccentric endurance training, especially in cardio rehabilitation, because this allows increasing the mechanical and metabolic stimulus to recruited muscle during the work intervals compared to continuous forms of exercise [25,26,27]. Using a soft robotic device that has been designed for neuromuscular rehabilitation, we have recently demonstrated that interval-type eccentric pedaling exercise at moderate intensity reproduces physiological feature being associated with both resistance, and endurance, type eccentric exercise stimulus, including the lower cardiovascular and aerobic cost than concentric interval exercise at a work-matched intensity, and the acutely elevated glucose concentration [19]. Subsequently, established methodology allowed for the real-time monitoring and quantification of mechanical and metabolic stress impacting on skeletal muscle during interval exercise [19,28]. However, the mechanism underlying the contraction type-dependent acute effects of interval type pedaling exercise, with specific reference to the impacting stimuli on the soft robot, and its suitability to produce contraction type-dependent compensatory cardiovascular and muscular reactions with repeated sessions of exercise, were not addressed. This yielded to the aim of the study to characterize the local and systemic effects of eccentric type of interval training compared to workload matched concentric interval exercise. The assumption was that indices of cardiovascular stress during exercise (as indicated by the rises in systolic blood pressure, blood glucose, and lactate concentration) is lowered to a different, possibly larger, degree after eccentric compared to concentric interval training when aerobic performance and non-aerobic strength are improved as well. As shown for continuous forms of exercise training, we reasoned that the observable physiological changes of aerobic muscle performance and metabolism stand in a linear relationship to the indices of mechanical and metabolic stress to which skeletal muscle and the heart are exposed during the exercise sessions (i.e., power output, muscle oxygen deficit, and hemoglobin concentration). 

## 2. Materials and Methods

### 2.1. Participants 

The sample size was calculated with prospective power analyses based on the effect sizes estimated from the published values for relevant parameters (Supplemental Table S1). The calculations showed that a minimal number of five subjects was required to prospectively identify (interaction) effects of protocol × training for the most important parameters. Thirteen subjects were recruited for this study upon having credited their written informed consent to participate in this investigation into the effects of concentric compared to eccentric interval-type of training on an Allegro soft robot (Dynamic Devices, Zurich, Switzerland) [19]. 

They had to meet the inclusion criteria of good health, such as not presenting a handicap that did not allow them to follow instructions (language barrier) and carry out the exercise such as musculoskeletal or cardiovascular injury or disease, and be of an age of between 45–70 years. Physical healthiness was assessed by a health questionnaire, and the absence of cardiovascular abnormalities was verified by a physician based on electrocardiographic measurement at rest and during the ramp-incremental exercise test with a 12-channel PC-ECG (customed, Ottobrunn, Germany). Two groups of six subjects each participated in either of the two training protocols. One subject had to be excluded due to an incapacity to perform the protocol of interval exercise satisfactorily. The training intervention was approved by the ethics committee of the canton of Zurich (Basec nr: 2016-02060) and registered on the clinical trials register (https://clinicaltrials.gov/ct2/show/NCT02845063). 

### 2.2. Study Design 

Twenty-four repeated sessions of progressive interval pedaling exercise under concentric or eccentric contractions were conducted over eight weeks. Cardiovascular and muscular parameters were assessed during interval pedaling exercise and a validated ramp test of cyclic pedaling before and after training. Aerobic performance was judged based on peak VO_2_ and peak aerobic power output (PPO) as determined with spiroergometric measurements during the ramp test. Anaerobic performance (i.e., leg muscle strength) was estimated based on measures of negative and positive peak power (NPP and PPP, respectively) of single contractions on the soft robot. Subjects reported on three occasions to the laboratory in the week prior to the commencement of training to carry out anthropometric measurements of weight and height, anaerobic performance, perform the ramp test, and conduct two familiarization sessions of interval pedaling exercise, being interspersed by two to three days of rest. Before and after the last training session, continuous measurements of metabolites in the blood (glucose and lactate concentration), cardiovascular parameters (heart rate, systolic and diastolic blood pressure), as well as oxygen saturation and hemoglobin concentration in exercised knee extensor muscle (as a measure of local metabolic stress), and the performed mechanical work were conducted during interval exercise. As well, before and after training, continuous measurements of blood metabolites and cardiovascular parameters were conducted during the ramp test. Subjects were assigned to the group training under a concentric or eccentric contraction protocol in random order. The target workload of the exercise was based on peak aerobic power as determined during the ramp test. All test sessions were repeated in the week after completion of the training. Each exercise session was carried out under the supervision of a qualified Sports scientist.

### 2.3. Soft Robotic Device 

The Allegro soft robot (Dynamic Devices, Zurich, Switzerland) functions as a dynamic leg press with haptic feedback, allowing to perform concentric as well as eccentric muscle work [19] at an adjustable target workload and with measures of the effective power output being generated. The robot was operated by pneumatic artificial muscles, and the pressure and range of length were software controlled and allowed imposing a load on both legs individually with a given contraction protocol. In this instance, this involved a modified version of the ‘concentric wheel’ or ‘eccentric wheel’ protocol of the Cardiometabolic-training module of the soft robot [19]. The robotic device also recorded the mechanical performance at a sampling rate of 200 Hz, allowing exporting the values of performed negative, positive, and average power and work. 

### 2.4. Training 

The training intervention consisted of repeated sessions of interval pedaling exercise on a soft robotic device (Allegro, Dynamic Devices, Zurich, Switzerland) under predominantly concentric or eccentric contractions at an aerobic peak power matched workload. Three training sessions were conducted per week for the eccentric, as well as the concentric exercise, under the Cardio Power training protocol of the robot essentially as described [28]. Each exercise session consisted of five minutes of rest while the feet remained on the pedal, followed by repeated intervals of one-minute of pedaling at 30 revolutions per minute (rpm) at a target power and a one-min cool-down period while feet were placed on the pedal. 

**Figure 1:**
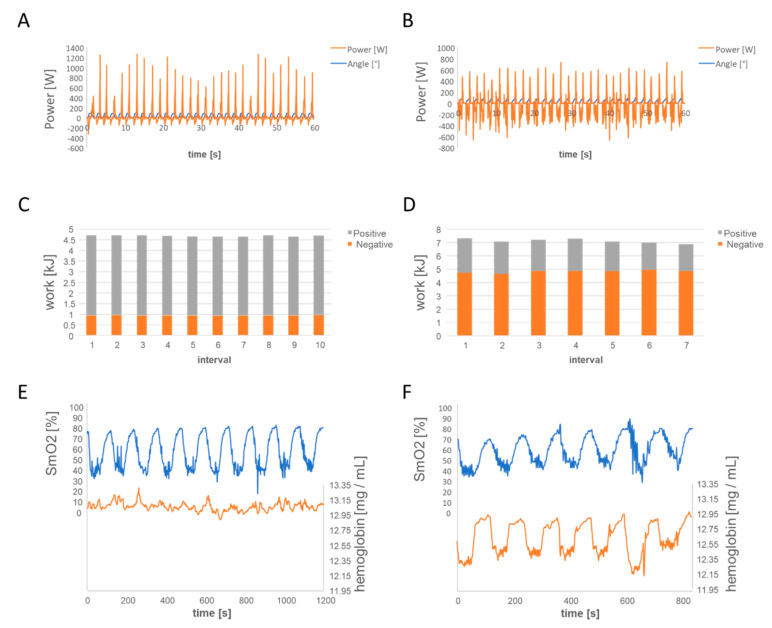
Power output and muscle oxygen saturation during workload-matched concentric and eccentric type of interval exercise. (**A**,**B**) Example of the power output being produced by one subject during one interval of concentric (**A**) and eccentric (**B**) interval exercise, respectively, before training. (**C**–**F**) Positive and negative work being performed by the left leg of one subject during all intervals of a session of concentric (**C**) and eccentric (**D**) interval exercise and the resulting effects on muscle oxygen saturation and hemoglobin concentration in m. vastus lateralis (**E**,**F**) for one leg before training.

The work to be conducted over all intervals of an exercise session was matched to reveal the same amount relative to PPO. For the eccentric protocol, the targeted power output was ~40% higher, and a correspondingly reduced number of intervals was performed to ascertain that the same work relative to PPO was performed per exercise session. The developed power and work produced per exercise session were monitored at the end of each two weeks phase of training and increased by raising the number of intervals or target power per session if subjects met the requested intensity and rate of force development. Table 1 summarizes the target power and number of intervals for each session of exercise in the four two-week-phases of training (Table 1). The protocol to be applied was optimized through adjustments in the targeted workload and software-based physical characteristics of the cycles of pneumatically applied load in previous investigations [19,28] to allow the application an optimal ratio of positive vs. negative work to be conducted (see Figure 1).

### 2.5. Test Sessions

Ramp Tests of exercise to exhaustion were conducted under concomitant cardiorespiratory measurements (cardiorespirometry) on an electrically-braked stationary cycle ergometer (Ergoselect 200, Ergoline, Bitz, Germany) where pedaling commenced after 3 min of rest with incremental increases in target workload essentially as described [19,28]. Familiarization sessions of interval pedaling exercises were conducted at the workload of the first session of interval exercise during the training. The post-training session of interval exercise on the soft robot was conducted at the workload of the last exercise session of the training. Peak power and the rate of force development of single negative and positive contractions were carried out on the soft robot for both legs essentially as described [19,28]. Measures of blood glucose and lactate concentrations, heart rate, systolic and diastolic blood pressure, and the estimation of the perceived rate of exhaustion were carried out essentially as described [19,28]. In brief, measurements started at rest and were repeated every two minutes after exercise was started. Blood was collected from the ear lobe (2 × 20 μL capillary blood). 20 μL of blood, each, was subjected to the quantification of glucose, using the OneTouch^®^ Vita™ Blood Glucose Monitoring System (Lifescan, Milpitas, CA, USA), and the measurement of lactate, using the Lactate Pro System (Axonlabs AG, Baden-Dättwil, Switzerland) during the interval exercise; or the Biosen C-line analyzer (EKF-diagnostic GmbH, Barleben, Germany) during the ramp test. Cardiovascular parameters were monitored with a pulse belt (Suunto, Vantaa, Finland) and a sonomed pulsoxymeter (Sonomed GMBH, Volketswil, Zürich). Systolic and diastolic blood pressure was assessed with blood pressure cuffs (SunTech Medical, North Carolina, USA) during the interval exercise or manually during the ramp test. 

### 2.6. Measurement of Oxygen Saturation and Hemoglobin Concentration

Measurements with near-infrared spectroscopy were conducted on Musculus vastus lateralis and m. gastrocnemius with a portable muscle oxygen monitor (Moxy, Fortiori Design LLC, Hutchinson, MN, USA) essentially as described [19,28]. The Moxy monitor uses four different light sources covering wavelengths in the range from 630 to 850 nm and the Beer-Lambert law to perform the quantitative measurements of muscle oxygen saturation and hemoglobin in the investigated voxel at 0.5–2 Hertz. Measurements were made from a shaved region of the left leg of the subjects. The sites of measurement situating for the m. vastus lateralis 10 cm above the upper lateral point of the patella along the major axis of the leg and for the m. gastrocnemius on a fictive line between the Malleolus medialis and medial plateau of the tibia. Data were exported via USB port to a portable computer using software MoxyPC and processed with MS-Excel (MS Office, Kildare, Ireland) to extract values for muscle oxygen saturation and hemoglobin concentration, and to calculate the oxygen deficit and hemoglobin concentration accruing during exercise as an area under the curve (AUC). 

### 2.7. Statistical Analysis 

Prospective and post-hoc power analyses were calculated using G*Power (G*Power 3.1.9.6, Heinrich-Heine-Universität Düsseldorf, Germany; http://www.gpower.hhu.de/) based on estimated effect sizes, and a α-level of 0.05, and a power (1-β) of 0.80. Data were curated using MS-Excel (Microsoft, Kildare, Ireland) and analyzed in SPSS (version 23, IBM Corporation, Armonk, NY, USA). Missing values for cardiovascular measurements were interpolated based on linear regression. Data are presented as mean ± standard deviation (SD) or standard error (SE). Effects on assessed metabolic parameters in the cardiovascular, blood and muscle, and mechanical output during interval exercise, as well as parameters of aerobic capacities during the ramp test, were assessed with a repeated-measures ANOVA for the between factors ‘contraction group’ (i.e., exercise under the eccentric/concentric contraction protocol) and ‘time’, the repeated (within) factor ‘training’ (pre/post), and where applicable muscle (m. vastus lateralis/m. gastrocnemius), with a correction for Roy’s square root and post-hoc tests of least significant difference. Linear relationships were calculated based on Pearson correlations. They were considered relevant if they passed thresholds of |r| > 0.70 and *p* < 0.05 and displayed using the cytoscape software 3.5.0 (https://cytoscape.org/) followed by subsequent ‘manual’ network analysis after curation of the connectivity in MS-Excel (MS Office, Kildare, Ireland). 

## 3. Results

### 3.1. Subjects

Table 2 summarizes the physiological characteristics of the participating subjects. Subjects demonstrated an average level of aerobic fitness. At baseline, no difference existed for any of the assessed parameters between the subjects that entered concentric or eccentric interval training.

### 3.2. Characteristics of the Muscle Stimulus during Interval Exercise

Figure 1 shows an example of the power being produced by the two groups during the duty cycles of one interval of exercise under the concentric and eccentric protocol, and the concomitant oxygen saturation and hemoglobin concentration in the contracting leg muscles m. vastus lateralis and m. gastrocnemius. The work per PPO to be produced during interval exercise did not differ between the two training groups before they began to exercise under the concentric and/or eccentric protocol (*p* = 0.509; Table 1). The contribution of negative and positive work being performed during each exercise session differed between the two groups conducting the aerobic power-matched eccentric or concentric contraction protocol (*p* < 0.001). The ratio of negative versus positive work being performed was 2.4-fold higher during eccentric than concentric exercise. Oxygen saturation in the leg muscles, m. vastus lateralis and m. gastrocnemius, was lowered with each set of concentric and eccentric interval exercise (Figure 1). Hemoglobin concentration in the two leg muscles was selectively lowered with eccentric exercise and accrued to a lower extent than during concentric exercise (Table 3).

### 3.3. Cardiovascular and Metabolic Reactions during Interval Exercise

**Figure 2:**
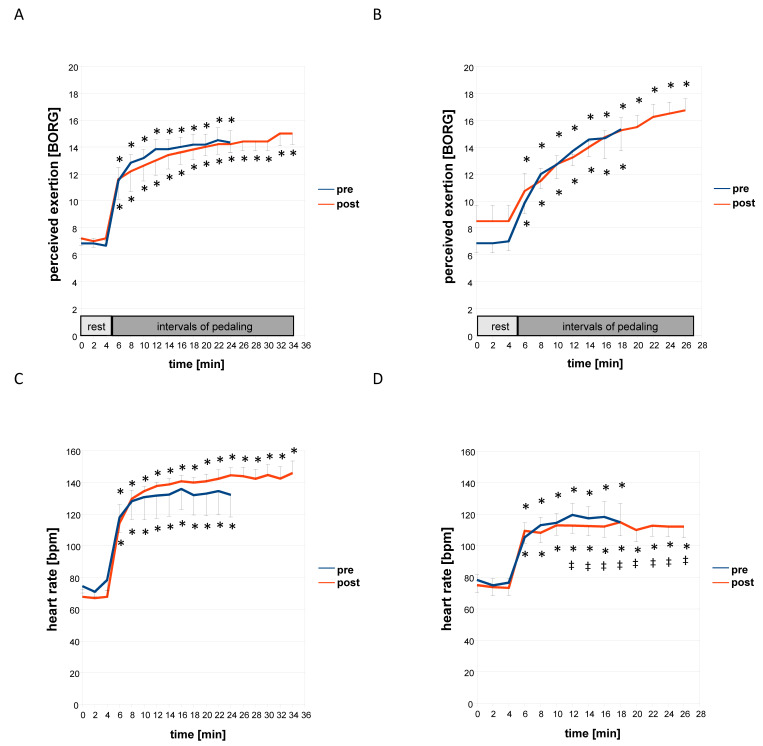
Temporal response of perceived exertion and heart rate during concentric and eccentric type of interval exercise. Line graph with whiskers indicating mean values ± SE for perceived exertion (**A**,**B**) and heart rate (**C**,**D**) as measured each 2 min during the interval-type pedaling exercise before and after the eight weeks of training of the two groups under the concentric (**A**,**C**) or eccentric (**B**,**D**) contraction protocol. The rest and pedaling phase of the respective concentric and eccentric interval exercise is exemplarily indicated in panels A and B. *, *p* < 0.05 vs. 0 min. ‡, *p* < 0.05 vs. concentric. Repeated-measures ANOVA with a post-hoc test of least significant difference.

**Figure 3:**
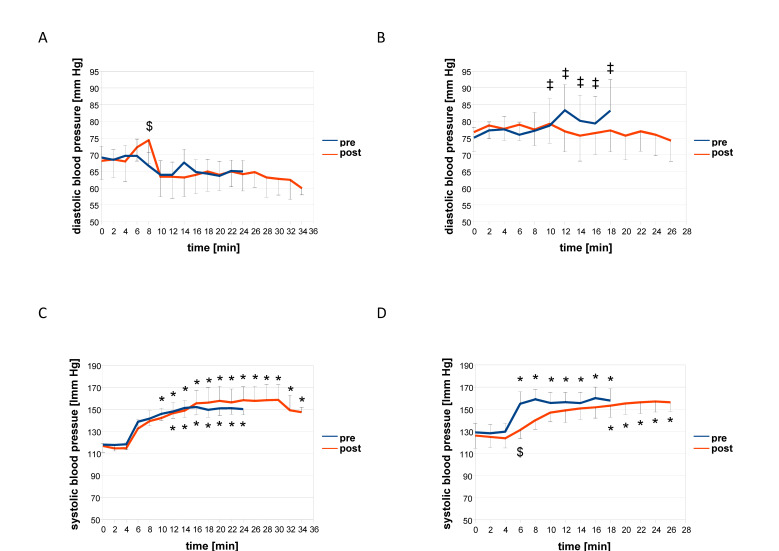
Temporal response of systolic and diastolic blood pressure during concentric and eccentric type of interval exercise. Line graph with whiskers indicating mean values ± SE for diastolic blood pressure (**A**,**B**) and systolic blood pressure (**C**,**D**) as measured each 2 min during the interval-type pedaling exercise before and after the eight weeks of training under the concentric (**A**,**C**) or eccentric (**B**,**D**) contraction group. *, *p* < 0.05 vs. 0 min. ‡, *p* < 0.05 vs. concentric. $ *p* < 0.05 vs. pre. Repeated-measures ANOVA with a post-hoc test of least significant difference.

**Figure 4:**
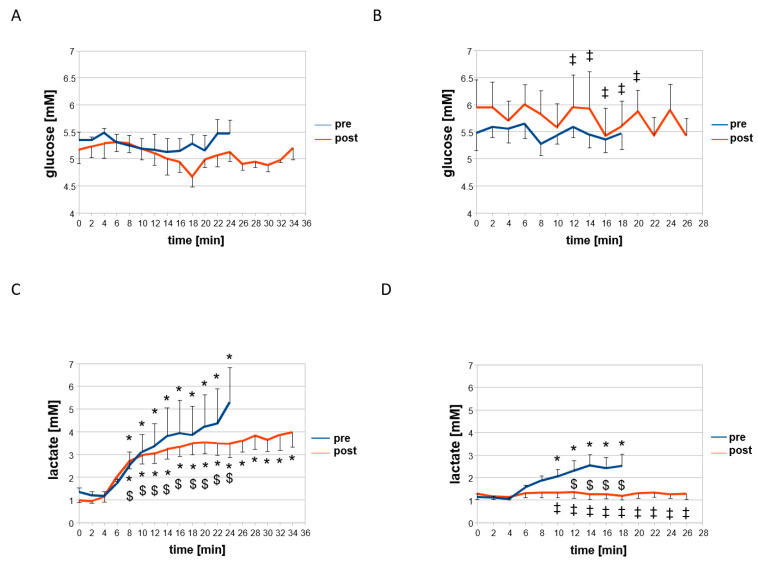
Temporal response of blood glucose and lactate concentration during concentric and eccentric type of interval exercise. Line graph with whiskers indicating mean values ± SE for blood glucose concentration (**A**,**B**) and blood lactate concentration (**C**,**D**) as measured each 2 min in the two groups during the interval-type pedaling exercise before and after the eight weeks of training under the concentric (**A**,**C**) or eccentric (**B**,**D**) contraction protocol. *, *p* < 0.05 vs. 0 min. ‡, *p* < 0.05 vs. concentric. $, vs. pre. Repeated-measures ANOVA with post-hoc test of least significant difference.

Figure 2, Figure 3 and Figure 4 and Table 3 and Table 4 summarize the acute consequences of a single session of concentric and eccentric exercise on metabolic and cardiovascular parameters. Prior to training, and independent of the contraction protocol, heart rate and the rate of the perceived exertion rose abruptly after the first 1 min interval of pedaling (minute six of the measurements, Figure 2). Systolic blood pressure and the blood concentration of lactate increased differently between the two groups exercising under the eccentric or concentric contraction protocol. Systolic blood pressure increased after the first and second interval of pedaling, i.e., minutes 6 and 8, during eccentric exercise but not during concentric exercise (Figure 3D). Blood lactate concentration was increased continuously and at earlier intervals during concentric than eccentric exercise. Diastolic blood pressure and the blood concentration of glucose were not affected at any time point during concentric or eccentric interval exercise (Figure 3A,B and Figure 4A,B).

Before training, differences revealed between the effects of eccentric and concentric exercise in the two groups of subjects for the time course of alterations in the total concentration of muscle hemoglobin (*p* < 0.001, Table 3), the perceived exertion (*p* = 0.013, Figure 2), systolic blood pressure (*p* = 0.019, Figure 3), and the blood concentration of glucose (*p* = 0.006, Figure 4). The latter three indices of metabolic stress were more elevated during the course of eccentric compared to concentric interval exercise by the two contraction groups when the total muscle hemoglobin concentration was lower during eccentric compared to concentric interval exercise (Table 3). Total and average oxygen deficit and hemoglobin concentration accruing during interval exercise did not differ between the two assessed muscle groups (Table 3).

### 3.4. Progression of the Imposed Exercise Stimulus with Interval Training

The imposed PPO-related target power (*p* = 0.001) and workload per exercise session (*p* < 0.001) increased progressively during the four phases of interval training (Table 1). In the last training phase, the power per PPO (*p* = 0.001) but the work per PPO (*p* = 0.644) being performed per exercise session did not differ between the two groups performing concentric or eccentric contractions. At the end of the eight weeks of training, 2.8-fold more negative than positive work was produced during eccentric exercise than during concentric exercise.

### 3.5. Training Modifies Metabolic and Cardiovascular Reactions during Interval Exercise

The course of alterations for indices of metabolic stress, i.e., the rate of perceived exertion, heart rate, systolic blood pressure, blood concentrations of lactate and glucose, the total oxygen deficit and average oxygen deficit in skeletal muscle, during concentric and eccentric exercise of the two groups were differently affected after interval training (Table 3 and Table 4). 

At the post-hoc level, the main effects resolved to a mitigated increase for the rate of perceived exertion (−9.6%), heart rate (−13.2%), systolic blood pressure (−6.4%), blood lactate concentration (−43.0%), and average oxygen deficit (−28%, *p* = 0.06), over the course of eccentric interval exercise after training after compared to before. In the group of subjects that had trained eccentrically, the rise in systolic blood pressure during eccentric exercise was delayed to later intervals (Figure 3). Conversely, systolic blood pressure (+4.0%) and total muscle oxygen deficit (+82%, Table 3) during concentric interval exercise were increased in the group of subjects that had trained concentrically compared to before training. Heart rate during interval exercise in the trained state (*p* = 0.001) was unlike in the untrained state (*p* = 0.373), more elevated under concentric than eccentric contractions in the respective contraction group. Blood glucose concentration was 3.3% lower during concentric interval exercise in the concentrically trained group, being lower during minutes 12 to 20 of the session than during eccentric interval exercise in the group that had trained eccentrically (Figure 4).

### 3.6. Training Modifies Metabolic and Cardiovascular Parameters before and after Interval Exercise

Before interval training, the subjects perceived the eccentric interval exercise as more exerting at the end of the last interval than the concentric interval exercise (Supplemental Figure S1). Blood glucose concentration at the end of eccentric and concentric interval exercise in the two respective contraction groups (*p* = 0.91, *p* = 0.82) and after the 4 min of rest did not differ significantly respective to the levels before exercise (*p* = 1.00, *p* = 0.50). Equally, blood glucose concentration during the 4 min of rest before exercise did not differ between the group of subjects training eccentrically and concentrically (*p* = 0.275). After the eight weeks of training, the blood glucose concentration during rest before the interval exercise was lower in those subjects that trained concentrically than those that did train eccentrically.

Heart rate declined more pronouncedly in the eccentrically- than concentrically-trained subjects during the four minutes of rest after the end of exercise. Blood lactate concentration was lower after eccentric than concentric exercise in the respective group after training, at the end, and during the first 4 min of rest after exercise.

### 3.7. Effects of Training and Contraction Protocol in the Two Groups on Aerobic Capacity and Power

Table 5 summarises the alterations in aerobic and anaerobic performance with interval training. There was an interaction effect of the training × contraction group for the positive peak power of single contractions and the respective rate of force development. At the post-hoc level, these effects were localized to an increased anaerobic positive peak power and rate of force development in the group that had trained eccentrically. The group that had trained concentrically demonstrated a trend (*p* = 0.084) for an increase in positive peak power. Negative peak power decreased after training in the eccentric contraction group. 

The values for peak oxygen uptake and peak power output as assessed in the ramp test were not affected by the eight weeks of training, irrespective of the contraction group. However, there was a trend for an increase in aerobic peak power output in the group that had trained under the concentric interval protocol (*p* = 0.060). 

### 3.8. Training and Contraction Protocol Modify Cardiovascular Reactions during Cyclic Ramp Exercise

Supplemental Figure S2 shows the temporal response of cardiovascular and metabolic indices of stress during the ramp test. Heart rate, systolic blood pressure, and the rate of perceived exertion were elevated 1 min after the commencement of pedaling, at test minute 4, before blood lactate concentration rose above resting levels. 

Table 6 summarises the interaction effects between interval training and the contraction group for the temporal response of metabolic/cardiovascular indices of stress during the ramp test. For the blood lactate concentration and heart rate during exercise, the main interaction effects were identified between training × contraction group. The effects on blood lactate concentration were explained by lower values during exercise in the group that had trained concentrically, and by increased values after interval training in the eccentric contraction group.

### 3.9. Relationships between the Muscle Stimulus and Adjustment of Cardiovascular/Metabolic Parameters

**Figure 5:**
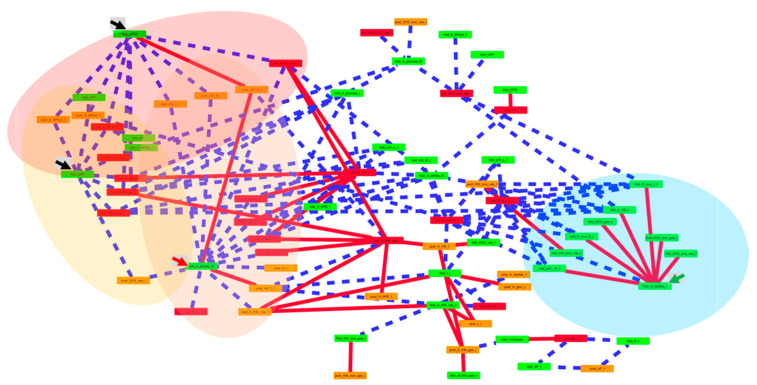
Connectivity of correlations between stress during the stimulus of interval exercise and the adjustments with training. Network display of the 131 linear relationships between indices of metabolic and mechanical stress during interval exercise and training-induced adjustments (nodes) for Pearson correlations, which passed a threshold of |r| > 0.70 and *p* < 0.05. Straight red and stippled blue lines, respectively, indicate positive and negative correlations. The strength of the correlation is indicated by the thickness of the lines connecting two nodes. Red and orange colored nodes highlight stress during the first and last session of interval exercise, respectively. Green colored nodes emphasize training-induced fold changes. Note the high connectivity with parameters demonstrating an interaction effect of interval training × the contraction group with the reddish-indicated indices of stress during interval exercise that define (highlighted) entities. This comprises selective correlations of the green-highlighted fold changes in positive peak power and the corresponding rate of force development (black arrows), the fold changes for the AUC of the blood lactate concentration during the ramp test (red arrow) or during interval exercise (green arrow), with the AUC of the perceived exertion, heart rate, the systolic blood pressure, the hemoglobin accruing in m. gastrocnemius, and the oxygen deficit in m. vastus lateralis, and the average power during interval exercise. For the ramp exercise, only the AUCs over the same duration of exercise before training were considered. Abbreviation code: _A, AUC during exercise; BPdia, diastolic blood pressure; BPsys, systolic blood pressure; bm, body mass; DO2, oxygen deficit; DO2_ave, average oxygen deficit; fold, post vs. pre ratio; gas, m. gastrocnemius; glucose, blood glucose concentration; HR, heart rate; _I, during interval exercise; L, _S, number of intervals (sets); left leg; lactate, blood lactate concentration; nPP, negative peak power; nW, negative work; P_ave, average power; post, after training; PPO, peak power output during the ramp test; pPP, positive peak power; pre, prior to training; pW, positive work; rPP, reactive peak power; R, right leg; _R, during ramp test; pRFD, rate of force development during the development of positive peak power; RPE, rate of perceived exertion; sP, target power per PPO; _t, exercise duration; tHb, concentration of total hemoglobin; vas, m. vastus lateralis; tHb_ave, average concentration of total hemoglobin; VO2peak, peak oxygen uptake.

One hundred-and-fifty linear relationships were identified between the fold differences post vs. before training for parameters, being measured during the ramp test and interval exercise, and the values for assessed indices of mechanical/metabolic muscle stress during interval pedaling exercise (Figure 5). For the training-induced (functional) adjustments that differed between the contraction groups/protocols, the highest connectivity of linear relationships revealed for the fold changes in peak power and the corresponding rate of force development, the time integral (AUC) of the fold changes in blood lactate concentration during the ramp test, as well as the fold changes in the AUCs for the blood concentration of lactate and glucose, and heart rate during interval exercise (Supplemental Table S2). On the other side, for the indices of the stress impacting during the first session of interval exercise, the highest connectivity was observed for the AUCs of perceived exertion, heart rate, systolic blood pressure, total hemoglobin in m. gastrocnemius, oxygen deficit in m. vastus lateralis, and the average power during interval exercise (Supplemental Table S3).

## 4. Discussion

Interval-type paradigms of physical training are a suitable venue for unfit subjects to lower risk factors of health, such as cardiovascular fitness and muscle strength [28]. However, it is not understood to what degree the observable functional adaptations are interrelated and reflect quantitative relationships to the mechanical and metabolic stimuli impacting during the repeated sessions of interval exercise [1,29,30]. In this perspective, we characterized acute effects of work-matched eccentric and concentric interval exercise on a soft robot on cardiovascular and aerobic muscle parameters and muscle performance before and after training in two groups of subjects. Indices of metabolic and mechanical stress during exercise were monitored in real-time with the deployed soft robotic device, a wearable NIRS device, and routine cardiovascular measures and assessed for the significance of the correlative relationship with physiological parameters. The goal was to qualify the deployed interval training protocols for their effect size and suitability for cardiovascular rehabilitation [27,28]. The observations highlight the suitability of the applied paradigm of soft robotic exercise to provoke cardiovascular and muscular adaptations in unfit but healthy subjects. Collectively, our manuscripts provide an example of how continuous measurements of physiological stress with a wearable device can assist routine sensory measurements, to substantiate subjective stress and expose dose-effect relationships of health-relevant biological systems. Prospectively, our observations indicate that real-time sensory feedback may serve to predict improvements in bodily function with the repeated exposure to the stress of exercise.

We identified that the applied eccentric interval training improved the strength of single muscle contractions, i.e., peak power and rate of force development of single positive contractions (Table 5). Conversely, the response of blood lactate concentration and heart rate during a standardized ramp test was not lowered after interval training in the group of subjects exercising eccentrically, when this was seen after interval training in the group that exercised concentrically (Supplemental Figure S2). However, adjustments of the temporal response of the blood lactate concentration, heart rate, systolic blood pressure, and the rate of perceived exertion were identified during eccentric interval exercise with interval training of the respective contraction group (Figure 2, Figure 3 and Figure 4). These alterations are beneficial for endurance performance, imply a contraction specific improvement of fitness. Our observations are consistent with the superior effects of eccentric compared to concentric type of continuous cycling type training on muscle strength in deconditioned subjects [31]. They support the contention that contraction-type dependent differences in metabolic strain exist between workload-matched shortening and lengthening type of interval type exercise [19]. 

We note that peak aerobic power output tended to demonstrate and increase with interval training only in the concentric contraction group (*p* = 0.08), but not in the eccentric contraction group (*p* = 0.43). For the eccentric contraction group, we even observed a worsening of the response of blood lactate concentration during the standard test of ramp exercise after eccentric interval training, i.e., an increase in the lactate concentration during exercise. Conversely, the response of blood lactate concentration during ramp exercise was substantially dimmed after interval training in the concentric contraction group (compare Supplemental Figures S1J and S2L). The accumulation of lactate in blood characterizes muscle’s capacity to handle aerobic metabolic strain during contraction [32]. The results, therefore, imply that the capacity of skeletal muscle to fuel energetic requirements during concentric contractions was reduced in the group that trained eccentrically. This may be reflective of a reduction in the volume density of mitochondria in m. vastus lateralis as reported to occur in coronary artery disease patients with (endurance-type) eccentric training [7]. 

Intriguingly, eccentric interval training mitigated the rise in blood lactate concentration during eccentric interval exercise in the respective contraction group despite the higher workload at which the latter session was conducted (Figure 4D). This effect is of interest in respect to effects on the average oxygen deficit in exercised muscle during interval exercise. NIRS-based (Near-infrared spectroscopy) measures of oxygen consumption serve like blood lactate concentration, which characterizes muscle’s capacity to handle aerobic metabolic strain during contraction, as a proxy for mitochondrial respiratory capacity [32]. In this respect, we found that the total muscle oxygen deficit during eccentric interval exercise was only lower after, but not before training, than during work-matched concentric interval exercise in the respective contraction groups (Table 3). This observation is consistent with the reportedly lower rise in the arterio-venous oxygen difference during eccentric than work-matched concentric endurance exercise of coronary artery disease patients after five weeks of training [33,34]. Collectively, the findings support the contention that (interval or continuous) endurance type eccentric exercise lowers the reliance on aerobic metabolism during the task of lengthening contractions. Specifically, interesting associations in this regard refer to the positive linear correlation between the training-induced fold changes in the AUC of blood lactate concentration and the training-induced fold changes in the average oxygen deficit in m. gastrocnemius, and m. vastus lateralis during interval exercise (Supplemental Table S2). This as well concerns the interaction effect between training and the contraction group for the average oxygen deficit during interval exercise (Table 3), which resolved as a trend (*p* = 0.06) for 28% lower values in the average oxygen deficit during eccentric interval exercise after compared to before training. Collectively, the present novel observations relate to the increased aerobic metabolic efficiency of eccentric compared to concentric types of contraction [20,32].

In respect to the identified metabolic alterations and relationships, it is relevant to consider that the ratio of negative versus positive work between eccentric and concentric was increased after compared to before training (i.e., 2.8-fold vs. 2.4-fold). Positive work mainly contributes to enhanced metabolic efficiency of contractions [6,11]. Intriguingly, we find that the training-induced fold changes in the positive work performed by the right and left leg correlated positively with the AUC for the concentration of blood lactate during the interval exercise (r = 0.918 and 0.841, *p* < 0.005). This hints that interval training reduced the contribution of positive work in eccentrically trained subjects, lowering metabolic stain during the duty cycles of eccentric interval exercise. It remains to be explored to what degree this involves altered neuromuscular coordination [33] in addition to the suggested reduction in muscle’s mitochondria content that was suggested by a selective increase in positive peak power (Table 5), and the worsened lactate response during ramp exercise after eccentric training.

The main theme of the results by the network analysis was that contraction protocol-specific alterations in the blood lactate concentration during exercise with training of the respective contraction group stood in negative linear relationships to parameters characterizing metabolic and mechanical stress to contracting extensor muscles during the initial stimulus of interval exercise prior to training. This concerned, for instance, the positive work performed by the left and right leg, the exercise duration, the number of sets of interval exercise, the rate of perceived exertion, and the AUC for the hemoglobin concentration in m. vastus lateralis; as well as the deficit in oxygen in m. vastus lateralis during interval exercise in the trained state (Figure 5 and Supplemental Table S2). Our observations emphasize that interval training-induced improvement in muscle’s local aerobic fitness stands alike for other training interventions, in inverse quantitative relationship to markers of the initial fitness state before training [4]. 

Intriguingly, the training-induced fold changes in the AUC for the concentration of blood lactate during interval exercise (and the ramp test) correlated negatively with the positive work performed by the left and right leg in the trained state and the training-induced fold changes in the average hemoglobin concentration in m. vastus lateralis (Supplemental Table S2). As well, the AUC for the hemoglobin concentration in m. vastus lateralis before training correlated negatively with the fold changes in the rate of positive force development. Hemoglobin concentration is a marker of muscle perfusion. Collectively the observations imply a possibly limiting contribution of mechano-related, restrictive factors in the vascular beds of the exercising muscles [2] to the possible gains in muscle’s aerobic metabolic fitness with the deployed interval training paradigm. 

The notion of the relationship between the mechanical influences in contracting muscles and cardiovascular parameters during exercise is supported by numerous significant correlations. For instance, the negative correlations between systolic and diastolic blood pressure, prior to and after training, and the fold changes in positive peak power and the corresponding rate of force development (Supplemental Table S2, Figure 5). Interestingly, also negative correlation revealed between the fold changes in positive peak power and the oxygen deficit in m. vastus lateralis prior to and after interval training. The integrated nature of the metabolic adaptations to the repeatedly imposed exercise stimulus on the soft robot is corroborated by negative correlations between the accruing oxygen deficit in exercising skeletal muscle and the training-induced fold changes for the AUC of heart rate during interval exercise. Collectively, the observations emphasize that training-induced variability in the adaptive changes in muscle’s contractile and metabolic properties (such as anaerobic power vs. aerobic capacity) with interval-training stood in inverse quantitative relationship to (cardiovascular) reactions in skeletal and cardiac muscle during the sessions of exercise. 

We noted that eight weeks of eccentric interval training was able to tackle cardiovascular limitations in unfit but healthy subjects. For instance, eccentric interval training mitigated the early surge in systolic blood pressure after the first interval of eccentric exercise in the respective group of subjects (Figure 3D). This adaptation took place despite the fact that exercise was carried out at a higher intensity. Concomitantly, the rise in diastolic blood pressure and blood lactate concentration during eccentric interval exercise was mitigated in the eccentric contraction group after training. These observations indicate that the capacity to handle metabolic stress during lengthening contractions was specifically improved in the respective subjects with eccentric training. It is not understood whether previously reported improvements in cardiovascular functioning with eccentric endurance training involve left ventricular adaptations or cardiovascular reactions outside the heart [34]. Correlations between systolic blood pressure during interval exercise and the training-induced fold changes in positive peak power (Supplemental Table S2) suggest that the involved process potentially involves obstructive mechanical constraints during the shortening phase of the duty cycle. Our observations support the notion that eccentric interval training reduced the potential risks of a cardiovascular event due to acute cardiac overload [34].

Upon inspection of the individual time points, we identified, however, that the contraction group (and protocol) dependent influence of interval training on time integral (i.e., AUC) of blood glucose concentration during interval exercise (Table 4) was explained by overall higher values during eccentric exercise in the trained state (Figure 4). This observation questions the suitability of the deployed eccentric exercise protocol to improve glucose handling during exercise. Eccentric muscle activity has been reported to acutely decrease non-insulin and insulin-mediated glucose uptake in rats [35,36,37,38]. Different to the suggestion from the effect of resistance exercise, blood glucose concentration was not elevated during the recovery from eccentric exercise in the untrained state, and this was similar to the concentration at rest not affected by eccentric training (Figure 2 and Figure 3, Supplemental Figure S1). Rather cardiovascular reactions, including the heart rate after exercise, and systolic blood pressure during the first interval of eccentric exercise, were modified in a contraction group-specific manner by interval type training. However, interestingly the AUC for the blood glucose concentration during interval exercise in the untrained state was strongly correlated to systolic blood pressure during exercise (r = 0.82) and negative work performed by either leg (r = 0.78 and 0.80) in the untrained state (Supplemental Table S3). These results suggest that muscle-related hemodynamic processes may explain the reduced glucose uptake after acute eccentric exercise [38]. However, we did not find evidence that the applied stimulus of eccentric interval exercise at moderate intensity, acutely or chronically, affected glucose handling as reported for resistance type training stimulus based on eccentric contractions at a higher intensity once a week [23,24]. These contrasting findings indicate that more research is required to resolve to which extent eccentric training-induced effects on blood glucose concentration depend on the intensity, training volume, and frequency of training. 

A limitation of our study was the rather low number of biological replicas of subjects with considerable baseline differences in aerobic fitness and age, weakening the strength of conclusions from a direct comparison of the two contraction protocols. However, owing to the prospective sample size calculations (Supplemental Table S1) and the repeated measures during the time course of exercise, anticipated interactions between the effect of training and the contraction protocol could be identified for a number of strength and skeletal and cardiac muscle-related parameters (Table 4). With the exception of systolic blood pressure with, and after, the first interval of work, this interaction effect could not be allocated to a single time point (Figure 2, Figure 3 and Figure 4). As expected, based on the prospective consultation of the literature and power analysis, we did not identify specific effects of interval training between the two contraction groups for parameters being related to systemic aerobic capacity. A post-hoc power analysis (Supplemental Table S4) indicated that false negative effects were controlled at a level of significance that is commonly/reasonably acceptable (i.e., a beta of 0.20; [39]). Exception concerned the average hemoglobin concentration in skeletal muscle, diastolic blood pressure, and negative peak power. For RPE, serum glucose concentration, which also demonstrated high *p*-values for beta (i.e., 0.48 and 0.44), we identified an interaction effect between contraction group × training; implying that for these factors, the possibility of a type II error can be formally excluded. However, the hypothesis cannot be rejected that the measurements of the time course of glucose concentration during and after interval exercise and training were confounded by the included subjects. For instance, the blood glucose concentration was overall lower for the subjects training eccentrically to those training concentrically before (*p* = 0.006) and after training (*p* < 0.001). This finding was mainly associated with one subject in the eccentric training group that demonstrated consistently higher blood concentrations of glucose. In fact, some of these values, such as the concentration of glucose, qualified for a pre-diabetic state [37] and was only moderately affected after training (i.e., 7.1 vs. 6.7 mmol/L). 

Another aspect to be considered regarding the possible interpretations is that the workload and number of intervals differed between the post- and pre-test sessions of interval exercise. However, we did not identify evidence for major differences in physiological variables of fitness between the groups training concentrically and eccentrically, respectively, before training (Table 2, Supplemental Figure S2, the findings of our study, therefore, have to be interpreted in relation to the respective fitness of the investigated subjects. As well the fact that the groups of subjects only conducted the test of interval-type exercise with the respective loading protocol that was applied in the training, limits the interpretation of effects. Aside from the specific influence of lengthening type contractions on the identified relationships between metabolic parameters, our observations may be specific for the selected task where exercise is carried out intermittently with phases of rests between the effective workout. 

## 5. Conclusions

Intensity-related, possibly contraction-specific, cardiovascular adaptations in skeletal and possibly cardiac muscle took place with moderate-intensity interval training. Concomitant effects on the peak power of extensor muscle groups during single shortening contractions support that the deployed protocol of eccentric interval training has its merits in improving anaerobic strength through processes that stood in negative relationship to parameters of muscle-bound aerobic metabolism and muscle perfusion.

The training-induced lowering of the surge in afterload with the onset of eccentric pedaling and heart rate emphasize that lengthening types of interval training (Figure 3) has its merits in producing favorable adaptations that alleviate cardiovascular risk factors during exercise, except for glucose handling during the workout. Meanwhile, the progressively increased workload with eccentric training was different to concentric training, unable to maintain the metabolic stress during eccentric interval exercise in the studied group of subjects. Based on the correlations between training-induced adaptations and indices of metabolic and mechanical stress, the target workload and intensity applied during both eccentric and concentric exercise appeared too low to produce substantial improvement in cardiorespiratory function that can be identified during a standardized ramp test of pedaling exercise to exhaustion.

## Figures and Tables

**Table 1:** Target workload during interval pedaling exercise. Mean ± standard error (SE) of the peak aerobic power output (PPO)-related target power, intervals, and PPO-related work to be performed by the group of subjects in the four phases of interval-type training under the concentric or eccentric contraction protocol. ANOVA with post-hoc test for the least significant difference.

Training Phase	Contraction Group	Target Power/PPO (W/W)	*p*-Value (Group)	Intervals (Number)	Work/PPO (kJ/W)	*p*-Value (Group)
week one to two	concentric	0.54 ± 0.06	0.025	10	0.33 ± 0.04	
	eccentric	0.71 ± 0.098		7	0.30 ± 0.04	0.509
week three to four	concentric	0.60 ± 0.03	0.004	15	0.54 ± 0.03	
	eccentric	0.87 ± 0.043		11	0.57 ± 0.03	0.535
week five to six	concentric	0.67 ± 0.03	0.002	15	0.60 ± 0.02	
	eccentric	0.95 ± 0.031		11	0.63 ± 0.02	0.580
week seven to eight	concentric	0.73 ± 0.02	0.001	15	0.65 ± 0.02	
	eccentric	1.02 ± 0.027		11	0.68 ± 0.02	0.644
group: p			<0.001			0.581
h2			0.558			0.010
phase: p			0.001			<0.001
h2			0.421			0.848
phase × p			0.639			0.755
group:h2			0.052			0.037

**Table 2:** Physiological characteristics at baseline. Mean ± SE of the assessed physiological parameters and peak oxygen uptake. Repeated-measures ANOVA with a post-hoc test of least significant difference. PPP, positive peak power; NPP, negative peak power, pHR; peak Heart rate, pVO_2_; peak VO2max; m, male; f, female.

	Age	Gender	Height	Mass	PPP	NPP	PPO	pHR	pVO_2_
	(years)	m/f	(cm)	(kg)	(Watt)	(Watt)	(Watt)	(bpm)	(mL O2 min^−1^ kg^−1^)
concentric:	31.4 ± 4.5	2/4	171.2 ± 4.4	67.7 ± 5.1	850.8 ± 158.7	344.2 ± 75.3	277.6 ± 34.1	179.0 ± 4.1	49.2 ± 5.5
eccentric:	43.6 ± 5.6	2/4	175.2 ± 3.6	67.2 ± 4.1	719.1 ± 177.4	429.9 ± 84.2	237.2 ± 34.1	172.2 ± 5.1	35.3 ± 6.2
*p*-value:	0.121		0.497	0.857	0.597	0.472	0.426	0.359	0.140

**Table 3:** Metabolic stress of muscle during interval exercise. Top) Mean ± SD, and post-hoc *p*-values, bottom) and *p*-values of the main effects of interval ‘training’, the contraction ‘group’, and muscle, on the average and total oxygen deficit and hemoglobin concentration accruing in *m. vastus lateralis* and *m. gastrocnemius* combined during interval exercise before and after training (i.e., week 1–2 vs. week 7–8). Repeated-measures ANOVA with multivariate test correction for Roy’s Largest Root. Underlined *p*-values were deemed significant.

		Total Oxygen Deficit		Average Oxygen Deficit		Total Hemoglobin		Average Hemoglobin	
		(%SmO2 × s)		average (%SmO2)		total (g dL^−1^ × s)		average (g dL^−1^)	
**before training**	**concentric**	39,282.8 ± 19,690.1		35.6 ± 16.5		14,260.7 ± 1544.1		13.1 ± 0.7	
	**eccentric**	32,555.4 ± 20,440.6		41.7 ± 26.2		10,243.7 ± 341.4		13.1 ± 0.4	
*p*-value (concentric vs. eccentric)			0.489		0.575		<0.001		0.815
			*p*-value (vs. before)		*p*-value (vs. before)		*p*-value (vs. before)		*p*-value (vs. before)
**after training**	**concentric**	71,152.2 ± 31,540.2	<0.001	41.6 ± 18.5	0.256	20,004.0 ± 2771.1	<0.001	11.7 ± 1.8	0.069
	**eccentric**	37,903.5 ± 25,932.6	0.517	30.1 ± 20.6	0.060	13,180.7 ± 3366	0.017	10.5 ± 2.7	0.004
*p*-value (concentric vs. eccentric)			0.029		0.259		<0.001		0.286
***p*-value (training)**		0.006		0.473		<0.001		0.002	
***p*-value (training × group)**		0.037		0.036		0.092		0.236	
***p*-value (group)**		0.096		0.782		<0.001		0.365	
***p*-value (muscle)**		0.640		0.643		0.821		0.880	

**Table 4:** Effects of training and contraction group on the response of cardiovascular and metabolic parameters to interval exercise. Test statistics (*p*-values and effect sizes) for the single and interaction effect of interval ‘training’ × the contraction ‘group’ on the temporal alterations of cardiovascular parameters during interval exercise. ANOVA with test correction for Roy’s Largest Root. RPE, rate of perceived exertion.

	RPE		Lactate		Glucose		Heart Rate		Diastolic Blood Pressure		Systolic Blood Pressure	
	(BORG)		(mM)		(mM)		(bpm)		(mmHg)		(mmHg)	
	*p*-Value	h2-Value	*p*-Value	h2-Value	*p*-Value	h2-Value	*p*-Value	h2-Value	*p*-Value	h2-Value	*p*-Value	h2-Value
time	<0.001	0.812	<0.001	0.496	0.950	0.060	<0.001	0.808	0.987	0.044	<0.001	0.438
training	0.004	0.099	0.001	0.130	0.638	0.003	0.092	0.039	0.500	0.006	0.405	0.009
group	0.004	0.102	0.001	0.141	<0.001	0.162	0.009	0.091	<0.001	0.215	0.012	0.077
time × training	0.031	0.234	0.523	0.128	0.524	0.124	0.999	0.030	0.977	0.050	0.948	0.061
time × group	0.710	0.073	0.694	0.078	0.996	0.020	0.242	0.143	0.995	0.021	1.000	0.003
training × group	0.015	0.072	0.050	0.050	0.031	0.058	0.009	0.092	0.200	0.021	0.001	0.131
time × training × group	0.393	0.108	0.811	0.064	0.972	0.033	0.796	0.070	0.966	0.035	0.799	0.063

**Table 5:** Alterations in performance with interval-type pedaling training. Top) Mean ± SE of the post vs. pre difference in percent for parameters of aerobic and anaerobic performance with interval type training, and bottom) *p*-values for the single and interaction effect of interval ‘training’ × the contraction group’. Abbreviations: nRFD, rate of negative force development; pRFD, rate of positive force development. pVO_2_, peak VO2max. Repeated-measures ANOVA with post-hoc test of least significant difference.

	pVO_2_	PPO	PPP	pRFD	NPP	nRFD
% Post vs. pre	(mL O_2_ min^−1^ kg^−1^)	(Watt)	(Watt)	(N s^−1^)	(Watt)	(N s^−1^)
concentric:	−0.65 ± 1.81	4.90 ± 2.26	7.64 ± 3.80	3.98 ± 5.43	26.69 ± 17.62	−3.90 ± 9.79
*p*-value:	0.729	0.062	0.084	0.488	0.174	0.702
eccentric:	2.07 ± 2.80	2.19 ± 2.65	27.80 ± 5.02	38.41 ± 8.13	−40.73 ± 15.77	−10.32 ± 11.21
*p*-value:	0.488	0.431	0.001	0.002	0.036	0.388
*p*-values training	0.888	0.083	<0.001	0.004	0.022	0.373
group:	0.149	0.333	0.829	0.691	0.591	0.692
training × group):	0.353	0.499	0.043	0.029	0.391	0.687

**Table 6:** Training-induced alterations in the cardiovascular/metabolic response during ramp exercise. *p*- and h2-value for the single and interaction effects of ‘training’ (pre/post) × contraction ‘group’ (concentric/eccentric) for time-related alterations of cardiovascular parameters during the ramp test of cycling exercise to voluntary exhaustion. Repeated-measures ANOVA with post-hoc test of least significant difference. The *p*-values for effects that were deemed significant are underlined.

	RPE		Lactate		Glucose		Heart Rate		Systolic Blood Pressure		Diastolic Blood Pressure	
	(BORG)		(mM)		(mM)		(bpm)		(mmHg)		(mmHg)	
	*p*-Value	h2-Value	*p*-Value	h2-Value	*p*-Value	h2-Value	*p*-Value	h2-Value	*p*-Value	h2-Value	*p*-Value	h2-Value
time	0.002	0.194	<0.001	0.804	0.019	0.118	<0.001	0.932	0.221	0.034	0.005	0.166
training	0.011	0.141	0.100	0.06	0.273	0.027	0.631	0.005	0.628	0.005	0.007	0.155
group	<0.001	0.889	<0.001	0.25	0.572	0.116	0.045	0.088	<0.001	0.713	0.854	0.069
time × training	0.326	0.163	0.006	0.349	0.843	0.071	0.697	0.096	0.792	0.08	0.362	0.152
time × group	0.765	0.072	0.032	0.26	0.943	0.037	0.347	0.136	0.992	0.017	0.481	0.113
training × group	0.261	0.029	<0.001	0.296	0.497	0.011	0.007	0.153	0.148	0.047	0.186	0.039
time × training × group	0.993	0.017	0.067	0.227	0.981	0.024	0.544	0.103	0.739	0.074	0.295	0.147

## Data Availability

Data is available on special request for qualified persons.

## Supplementary Materials

The following are available online at https://www.mdpi.com/1424-8220/21/1/173/s1, Figure S1: Influence of training on metabolic and cardiovascular parameters before and after interval exercise, Figure S2: Temporal response of cardiovascular indices of metabolic stress during ramp exercise, Table S1: Sample size calculations. Table S2: Correlations between stress during the stimulus of interval exercise and the adjustments with training. Table S3: Correlations between stress during the stimulus of interval exercise. Table S4: Retrospective power analysis.
