# Supplementary material for: The Cardiovascular Response to Interval Exercise Is Modified by the Contraction Type and Training in Proportion to Metabolic Stress of Recruited Muscle Groups
Source: Sensors (Basel). 2020 Dec 29;21(1):173. doi: 10.3390/s21010173 (PMC7795051; doi:10.3390/s21010173)
Supplement: Supplementary file 1 [file sensors-21-00173-s001.pdf]

**Supplemental table 1:** *Sample size calculations.* Summary of the prospective power analysis on anticipated interaction effects between the contraction type (i.e. eccentric vs. concentric) x exercise/training on the exercise response that were of interest to our investigation. Calculations were carried out with G*Power with the indicated values for alpha and beta.

| **response type** | **parameter type** | **parameter** | **time point vs. exercise** | **exercise type** | **intensity per 1RM** | **exercise volume** | **contraction number** | **exercise device** | **muscle group** | **effect size f** | **alpha** | **power (1-beta)** | **sample size** | **references** | |
|---|---|---|---|---|---|---|---|---|---|---|---|---|---|---|---|
| acute | cardiopulmonary | peak cardiac output | during | interval type | 17% | 1x | 450 | soft robot | knee extensors | 0.913 | 0.05 | 0.80 | 16 | Fluck et al 2017 | |
| acute | cardiopulmonary | peak heart rate | during | interval type | 17% | 1x | 450 | soft robot | knee extensors | 1.214 | 0.05 | 0.80 | 10 | Fluck et al 2017 | |
| acute | cardiopulmonary | VO2peak | during | interval type | 17% | 1x | 450 | soft robot | knee extensors | 1.667 | 0.05 | 0.80 | 6 | Fluck et al 2017 | |
| acute | cardiopulmonary<br>Meyer et al 2003 | oxygen uptake | during | continuous cycling | | 30% | 1x, into training | 1650 | cycle ergometer | knee extensors | | 2.322 | 0.05 | 0.80 | 6 |
| acute | cardiopulmonary<br>Meyer et al 2003 | heart rate | during | continuous cycling | | 30% | 1x, into training | 1650 | cycle ergometer | knee extensors | | 0.818 | 0.05 | 0.80 | 12 |
| acute<br>1Mio | cardiovascular<br>Meyer et al 2003 | mean arterial blood pressure | during | continuous cycling | | 30% | 1x, into training | 1650 | cycle ergometer | knee extensors | | 0.115 | 0.05 | 0.80 | > |
| acute | cardiovascular<br>Meyer et al 2003 | systemic vascular resistance | during | continuous cycling | | 30% | 1x, into training | 1650 | cycle ergometer | knee extensors | | 0.395 | 0.05 | 0.80 | 38 |
| acute | cardiovascular<br>Meyer et al 2003 | arterio-venous O2 difference | during | continuous cycling | | 30% | 1x, into training | 1650 | cycle ergometer | knee extensors | | 1.570 | 0.05 | 0.80 | 6 |
| acute | muscle metabolism<br>0.80 | <br>12 | blood lactate concentration<br>Meyer et al 2003 | | during | continuous cycling | 30% | 1x, into training | 1650 | cycle ergometer | | knee extensors | 0.827 | 0.05 | |
| acute | muscle metabolism<br>0.80 | <br>15 | maximal blood lactate<br>Fluck et al, 2017 | | during | interval type | 17% | 1x | 450 | soft robot | knee extensors | | 0.941 | 0.05 | |
| acute | muscle metabolism<br>Fluck et al 2017 | | lactate finish | end | interval type | 17% | 1x | 450 | soft robot | knee extensors | | 2.000 | 0.05 | 0.80 | 5 |
| acute | muscle metabolism<br>Fluck et al 2017 | | lactate 8-min post | 8 min post | interval type | 17% | 1x | 450 | soft robot | knee extensors | | 2.135 | 0.05 | 0.80 | 5 |
| acute | cardiovascular | blood glucose concentration | end | interval type | 17% | 1x | 450 | soft robot | knee extensors | 0.385 | 0.05 | 0.80 | 62 | Fluck et al 2017 | |
| acute | cardiovascular | blood glucose concentration | 8 min post | interval type | 17% | 1x | 450 | soft robot | knee extensors | 0.308 | 0.05 | 0.80 | 94 | Fluck et al 2017 | |
| acute<br>2011 | cardiovascular | blood glucose concentration | 48 hrs post | resistance | 100% | 1x | 75 | dynamometer | knee extensors | 0.920 | 0.05 | 0.80 | 10 | Paschalis et al | |
| acute<br>2011 | muscle strength | peak torque | end | resistance | 100% | 1x | 75 | dynamometer | knee extensors | 1.028 | 0.05 | 0.80 | 8 | Paschalis et al | |

| | | | | | | | | | | | | | | | |
|---|---|---|---|---|---|---|---|---|---|---|---|---|---|---|---|
| acute<br>2011 | muscle strength | peak torque | 48 hrs post | resistance | 100% | 1x | 75 | dynamometer | knee extensors | 4.850 | 0.05 | 0.80 | 4 | Paschalis et al | |
| training | cardiopulmonary<br>12736 | VO2peak<br>Meyer et al, 2003 | at rest | continuous cycling | | 30% | 24 x over 8 weeks | 39600 | cycle ergometer | knee extensors | | 0.002 | 0.05 | 0.80 | |
| training<br>2011 | cardiovascular | blood glucose concentration | before & 48 h post | resistance | 100% | 8x over 8 weeks | 600 | dynamometer | knee extensors | 0.780 | 0.05 | 0.80 | 12 | Paschalis et al | |
| training | cardiovascular | blood glucose concentration | fasted at rest | leg extension | 75% | 12 x over 12 weeks | 540 | leg extension | knee extensors | 2.000 | 0.05 | 0.80 | 6 | Chen et al 2017 | |
| training | cardiovascular | HOMA index | at rest | leg extension | 75% | 12x over 12 weeks | 540 | leg extension | knee extensors | 3.400 | 0.05 | 0.80 | 4 | Chen et al 2017 | |
| training<br>2006 | muscle strength | one repetition maximum (1RM) | | at rest | resistance | 100% | 24x over 8 weeks | 1200 | arm curl | arms | 0.950 | 0.05 | 0.80 | 12Okamoto et | |
| training<br>1998 | muscle strength | one repetition maximum | at rest | hamstring curls | 100% | 12x over 6 weeks | 192 | leg-curl machine | hamstrings | 1.150 | 0.05 | 0.80 | 8 | Kaminski et al | |
| training | muscle strength | one repetition maximum | at rest | hamstring curls | 100% | 24x over 8 weeks | 360 | leg-curl machine | hamstrings | 1.290 | 0.05 | 0.80 | 6 | Potier et al 2009 | |
| training | muscle strength | peak torque | at rest | resistance | 100% | 60x over 20 weeks | 2400 | dynamometer | knee extensors | 0.920 | 0.05 | 0.80 | 10 | Seger et al 1998 | |
| training<br>2007 | muscle strength | torque | at rest | resistance | 100% | 30 x over 10 weeks | 900 | dynamometer | knee extensors | 0.290 | 0.05 | 0.80 | 86 | Blazevich etal | |
| training<br>1Mio | muscle strength<br>Meyer et al 2003 | PPO | at rest | continuous cycling | | 30% | 24x over 8 weeks | 39600 | cycle ergometer | knee extensors | | 0.000 | 0.05 | 0.80 | > |
| training | muscle strength<br>1976 | PPO<br>Meyer et al 2003 | at rest | continuous cycling | | 30% | 24x over 8 weeks | 39600 | cycle ergometer | knee extensors | | 0.050 | 0.05 | 0.80 | |

## References


Blazevich AJ, Cannavan D, Coleman DR, Horne, S. Influence of concentric and eccentric resistance training on architectural adaptation in human quadriceps muscles. J Appl Physiol 103: 1565– 1575, 2007.

Chen TCC, Tseng WC, Huang GL, Chen HL, Tseng KW, Nosaka K. Superior Effects of Eccentric to Concentric Knee Extensor Resistance Training on Physical Fitness, Insulin Sensitivity and Lipid Profiles of Elderly Men. Front Physiol 8: 209, 2017.

Fluck M, Bosshard R, Lungarella M, Cardiovascular and Muscular Consequences of Work-Matched Interval-Type of Concentric and Eccentric Pedaling Exercise on a Soft Robot, 8:640, 2017

Fluck M, Bosshard R, Lungarella M. Cardiovascular and Muscular Consequences of Work-Matched Interval-Type of Concentric and Eccentric Pedaling Exercise on a Soft Robot, 8:640, 2017.

Kaminski TW, Wabbersen CV, Murphy RM. Concentric versus enhanced eccentric hamstring strength training: clinical implications. J Athl Train 33(3): 216–221, 1998.

Meyer K, Steiner R, LaStayo P, Lippuner K, Allemann Y, Eberli F, Schmid J, Saner H, Hoppeler H. Eccentric Exercise in Coronary Patients: Central Hemodynamic and Metabolic Responses, Medicine & Science in Sports & Exercise 35(7) : 1076-1082, 2003.

Okamoto T, Masuhara M, Ikuta K, Effects of eccentric and concentric resistance training on arterial stiffness. J Hum Hypertens 20(5): 348-54, 2006.

Paschalis V, Nikolaidis MG, Theodorou AA, Panayiotou G, Fatouros IG, Koutedakis Y, Jamurtas AZ. A weekly bout of eccentric exercise is sufficient to induce health-promoting effects. Med Sci Sports Exerc 43(1): 64-73, 2011.

Potier TG, Alexander CM, Seynnes OR. Effects of eccentric strength training on biceps femoris muscle architecture and knee joint range of movement. Eur J Appl Physiol 105(6): 939–944, 2009.

Seger, JY, Arvidsson, B, and Thorstensson, A. Specific effects of eccentric and concentric training on muscle strength and morphology in humans. Eur J Appl Physiol Occup Physiol 79: 49–57, 1998.

***Supplemental table 2:*** *Correlations between stress during the stimulus of interval exercise and the adjustments with training.*

List of the 131 linear relationships between indices of metabolic and mechanical stress during interval exercise and training-induced adjustments (nodes) for Pearson correlations which passed a threshold of |r| >0.70 and p<0.05 as shown in a condensed manner in figure 5. Underlined parameters reflect those which demonstrated significant interaction effects of interval 'training' x the contraction 'protocol'. Abbreviations/code: _A, AUC during exercise; BPdia, diastolic blood pressure; BPsys, systolic blood pressure; bm, body mass; DO2, oxygen deficit; DO2_ave, average oxygen deficit; fold, post vs. pre ratio; gas, m. gastrocnemius; glucose, blood glucose concentration; HR, heart rate; _I, during interval exercise; _S, number of intervals (sets); _L, left leg; lactate, blood lactate concentration; nPP, negative peak power; nW, negative work; P_ave, average power; post, after training; PPO, peak power output

during the ramp test; pPP, positive peak power; pre, prior to training; pW, positive work; rPP, reactive peak power; R, right leg; _R, during ramp test; pRFD, rate of force development during the development of positive peak power; RPE, rate of perceived exertion; sP, target power per PPO; _t, exercise duration; tHb, concentration of total hemoglobin; vas, m. vastus lateralis; tHb_ave, average concentration of total hemoglobin; VO2peak, peak oxygen uptake.

| ***node_1*** | ***node_2*** | ***r-value*** |
|---|---|---|
| fold_tHb_ave_vas_I | fold_A_lactate_I | -0.785 |
| post_A_BPdia_I | fold_pPP | -0.701 |
| post_A_BPdia_I | fold_pRFD | -0.705 |
| post_A_BPdia_I | fold_rPP | -0.723 |
| post_A_BPdia_I | fold_tHb_ave_vas_I | -0.874 |
| post_A_BPsys_I | fold_pPP | -0.772 |
| post_A_BPsys_I | fold_pRFD | -0.816 |
| post_A_lactate_I | fold_tHb_ave_gas_I | -0.67 |
| post_A_tHb_gas_I | fold_VO2peak | -0.709 |
| post_A_tHb_vas_I | fold_A_lactate_R | -0.78 |
| post_DO2_ave_vas_I | fold_A_glucose_R | -0.738 |
| post_DO2_vas_I | fold_A_lactate_R | -0.789 |
| post_DO2_vas_I | fold_pPP | -0.699 |
| post_S_I | fold_A_lactate_R | -0.706 |
| post_nW_L_I | fold_A_tHb_vas_I | -0.693 |
| post_nW_L_I | fold_t_I | -0.732 |
| post_pW_L_I | fold_A_lactate_R | -0.736 |
| post_pW_L_I | fold_pRFD | -0.678 |
| post_pW_R_I | fold_A_lactate_R | -0.742 |
| post_pW_R_I | fold_pRFD | -0.689 |
| post_sP_I | fold_P_I | -0.772 |

| | | |
|---|---|---|
| post_sP_I | fold_sP_I | -0.767 |
| post_tHb_ave_vas_I | fold_A_BPdia_R | -0.698 |
| post_tHb_ave_vas_I | fold_A_HR_I | -0.678 |
| post_tHb_ave_vas_I | fold_P_ave_L_I | -0.686 |
| post_tHb_ave_vas_I | fold_P_ave_R_I | -0.7 |
| post_tHb_ave_vas_I | fold_pW__R_I | -0.697 |
| post_tHb_ave_vas_I | fold_pW_L_I | -0.67 |
| pre_A_BPdia_I | fold_A_BPdia_I | -0.696 |
| pre_A_BPdia_I | fold_bm | -0.841 |
| pre_A_BPdia_I | fold_pPP | -0.729 |
| pre_A_BPdia_I | fold_pRFD | -0.711 |
| pre_A_BPsys_I | fold_A_glucose_I | -0.829 |
| pre_A_BPsys_I | fold_nW_L_I | -0.697 |
| pre_A_BPsys_I | fold_nW_R_I | -0.676 |
| pre_A_BPsys_I | fold_pPP | -0.974 |
| pre_A_BPsys_I | fold_pRFD | -0.973 |
| pre_A_glucose_I | fold_A_glucose_I | -0.673 |
| pre_A_glucose_I | fold_pPP | -0.758 |
| pre_A_glucose_I | fold_pRFD | -0.848 |
| pre_A_HR_I | fold_A_BPdia_R | -0.735 |
| pre_A_HR_I | fold_A_glucose_I | -0.679 |
| pre_A_HR_I | fold_A_lactate_R | -0.715 |
| pre_A_HR_I | fold_A_RPE_I | -0.678 |
| pre_A_RPE_I | fold_A_BPdia_R | -0.751 |
| pre_A_RPE_I | fold_A_glucose_I | -0.746 |
| pre_A_RPE_I | fold_A_lactate_R | -0.706 |
| pre_A_RPE_I | fold_A_RPE_I | -0.677 |
| pre_A_RPE_I | fold_nW_L_I | -0.708 |
| pre_A_tHb_gas_I | post_nW_L_I | -0.85 |
| pre_A_tHb_gas_I | post_nW_R_I | -0.86 |

| | | |
|---|---|---|
| pre_A_tHb_vas_I | fold_A_BPdia_R | -0.709 |
| pre_A_tHb_vas_I | fold_A_lactate_R | -0.821 |
| pre_A_tHb_vas_I | fold_pRFD | -0.698 |
| pre_DO2_ave_gas_I | fold_A_glucose_R | -0.811 |
| pre_DO2_ave_vas_I | fold_A_BPsys_I | -0.695 |
| pre_DO2_ave_vas_I | fold_A_glucose_R | -0.868 |
| pre_DO2_ave_vas_I | fold_A_HR_I | -0.707 |
| pre_DO2_ave_vas_I | fold_nPP | -0.702 |
| pre_DO2_ave_vas_I | fold_P_ave_L_I | -0.671 |
| pre_DO2_gas_I | fold_A_glucose_R | -0.732 |
| pre_DO2_gas_I | fold_pPP | -0.671 |
| pre_DO2_vas_I | fold_A_glucose_R | -0.792 |
| pre_DO2_vas_I | fold_A_HR_I | -0.684 |
| pre_DO2_vas_I | fold_nW_R_I | -0.692 |
| pre_DO2_vas_I | fold_P_ave_L_I | -0.674 |
| pre_DO2_vas_I | fold_pPP | -0.697 |
| pre_S_I | fold_A_lactate_R | -0.732 |
| pre_nW_R_I | fold_A_tHb_vas_I | -0.7 |
| pre_nW_R_I | fold_t_I | -0.746 |
| pre_P_ave_L_I | fold_A_HR_I | -0.794 |
| pre_P_ave_L_I | fold_DO2_vas_I | -0.694 |
| pre_P_ave_L_I | fold_P_ave_L_I | -0.833 |
| pre_P_ave_L_I | fold_P_ave_R_I | -0.797 |
| pre_P_ave_L_I | fold_pW__R_I | -0.755 |
| pre_P_ave_L_I | fold_pW_L_I | -0.832 |
| pre_P_ave_L_I | fold_t_I | -0.768 |
| pre_P_ave_R_I | fold_A_BPdia_R | -0.702 |
| pre_P_ave_R_I | fold_A_HR_I | -0.826 |
| pre_P_ave_R_I | fold_DO2_vas_I | -0.676 |
| pre_P_ave_R_I | fold_nW_R_I | -0.692 |

| | | |
|---|---|---|
| pre_P_ave_R_I | fold_P_ave_L_I | -0.866 |
| pre_P_ave_R_I | fold_P_ave_R_I | -0.833 |
| pre_P_ave_R_I | fold_pW__R_I | -0.782 |
| pre_P_ave_R_I | fold_pW_L_I | -0.852 |
| pre_P_ave_R_I | fold_t_I | -0.706 |
| pre_P_I | fold_A_BPdia_R | -0.73 |
| pre_pW_L_I | fold_A_BPdia_R | -0.917 |
| pre_pW_L_I | fold_A_lactate_R | -0.838 |
| pre_pW_L_I | fold_nW_L_I | -0.687 |
| pre_pW_R_I | fold_A_BPdia_R | -0.896 |
| pre_pW_R_I | fold_A_lactate_R | -0.825 |
| pre_sP_I | fold_P_I | -0.959 |
| pre_sP_I | fold_sP_I | -0.957 |
| pre_t_I | fold_A_lactate_R | -0.732 |
| fold_A_HR_I | fold_A_lactate_I | 0.857 |
| fold_A_lactate_R | post_nW_R_I | 0.695 |
| fold_DO2_ave_gas_I | fold_A_lactate_I | 0.771 |
| fold_DO2_ave_vas_I | fold_A_lactate_I | 0.675 |
| fold_DO2_gas_I | fold_A_lactate_I | 0.809 |
| fold_P_ave_L_I | fold_A_lactate_I | 0.815 |
| fold_P_ave_R_I | fold_A_lactate_I | 0.872 |
| fold_pW__R_I | fold_A_lactate_I | 0.918 |
| post_A_gluc_I | fold_t_I | 0.768 |
| post_A_HR_I | fold_DO2_vas_I | 0.677 |
| post_A_HR_I | fold_t_I | 0.681 |
| post_A_tHb_gas_I | fold_A_tHb_gas_I | 0.722 |
| post_A_tHb_gas_I | fold_A_tHb_vas_I | 0.725 |
| post_A_tHb_gas_I | fold_t_I | 0.745 |
| post_A_tHb_vas_I | fold_A_tHb_vas_I | 0.723 |
| post_A_tHb_vas_I | fold_t_I | 0.702 |

| | | |
|---|---|---|
| post_nW_L_I | fold_A_lactate_R | 0.686 |
| post_nW_R_I | fold_pRFD | 0.7 |
| post_t_I | fold_A_tHb_vas_I | 0.718 |
| post_t_I | fold_t_I | 0.783 |
| post_tHb_ave_gas_I | fold_tHb_ave_gas_I | 0.963 |
| post_tHb_ave_vas_I | fold_tHb_ave_vas_I | 0.802 |
| pre_A_glucose_I | pre_A_HR_I | 0.759 |
| pre_A_RPE_I | pre_A_HR_I | 0.865 |
| pre_A_tHb_gas_I | post_A_HR_I | 0.758 |
| pre_A_tHb_gas_I | post_A_RPE_I | 0.763 |
| pre_A_tHb_gas_I | post_A_tHb_vas_I | 0.821 |
| pre_A_tHb_gas_I | pre_A_BPsys_I | 0.707 |
| pre_A_tHb_gas_I | pre_A_RPE_I | 0.827 |
| pre_A_tHb_gas_I | pre_A_tHb_vas_I | 0.984 |
| pre_A_tHb_gas_I | pre_t_I | 0.966 |
| pre_A_tHb_vas_I | pre_A_HR_I | 0.819 |
| pre_P_I | fold_PPO | 0.693 |
| pre_pW_L_I | pre_A_HR_I | 0.863 |
| pre_pW_R_I | pre_A_HR_I | 0.882 |
| pre_sP_I | fold_VO2peak | 0.705 |

***Supplemental table 3:*** *Correlations between stress during the stimulus of interval exercise.*

List of the 266 linear relationships between indices of metabolic and mechanical stress during interval exercise prior and post interval

training (nodes) for Pearson correlations which passed a threshold of |r| >0.70 and p<0.05. Abbreviations/code: _A, AUC during exercise; BPdia, diastolic blood pressure; BPsys, systolic blood pressure; bm, body mass; DO2, oxygen deficit; DO2_ave, average oxygen deficit; gas, m. gastrocnemius; glucose, blood glucose concentration; HR, heart rate; _L, left leg; lactate, blood lactate concentration; nW, negative work; P_ave, average power; post, after training; PPO, peak power output during the ramp test; pre, prior to training; pW, positive work; R, right leg; RPE, rate of perceived exertion; S, number of intervals (sets); sP, target power per PPO; t, exercise duration; tHb, concentration of total hemoglobin; vas, m. vastus lateralis; tHb_ave, average concentration of total hemoglobin.

| ***node_1*** | ***node_2*** | ***r-value*** |
|---|---|---|
| DO2_ave_gas_post | DO2_ave_vas_post | 0.679 |
| DO2_ave_gas_post | DO2_vas_post | 0.684 |
| DO2_ave_gas_pre | DO2_ave_vas_post | 0.793 |
| DO2_ave_gas_pre | DO2_ave_vas_pre | 0.869 |
| DO2_ave_gas_pre | DO2_vas_pre | 0.820 |
| DO2_ave_vas_post | DO2_ave_vas_pre | 0.842 |
| DO2_ave_vas_post | ave_tHb_vas_pre | 0.771 |
| DO2_ave_vas_post | DO2_gas_pre | 0.811 |
| DO2_ave_vas_pre | ave_tHb_vas_pre | 0.686 |
| ave_tHb_gas_pre | ave_tHb_vas_post | 0.696 |
| ave_tHb_gas_pre | ave_tHb_vas_pre | 0.778 |

| | | |
|---|---|---|
| ave_tHb_gas_pre | P_week3_4 | 0.840 |
| ave_tHb_gas_pre | P_week5_6 | 0.868 |
| ave_tHb_vas_post | ave_tHb_vas_pre | 0.691 |
| ave_tHb_vas_post | P_ave_L_pre | 0.741 |
| ave_tHb_vas_pre | P_ave_L_pre | 0.675 |
| ave_tHb_vas_pre | P_ave_R_pre | 0.716 |
| ave_tHb_vas_pre | DO2_gas_pre | 0.734 |
| ave_tHb_vas_pre | DO2_vas_post | 0.701 |
| ave_tHb_vas_pre | P_week5_6 | 0.683 |
| ave_tHb_vas_pre | P_week7_8 | 0.728 |
| ave_tHb_vas_pre | pW_L_pre | 0.740 |
| ave_tHb_vas_pre | pW_R_pre | 0.709 |
| P_ave_L_post | P_ave_R_post | 0.895 |
| P_ave_L_pre | P_ave_R_pre | 0.984 |
| P_ave_R_post | P_week1_2 | 0.680 |
| P_ave_R_post | P_week3_4 | 0.722 |
| P_ave_R_post | P_week5_6 | 0.744 |
| P_ave_R_post | P_week7_8 | 0.753 |
| P_ave_R_pre | ave_tHb_vas_post | 0.810 |

| | | |
|---|---|---|
| P_ave_R_pre | P_week7_8 | 0.673 |
| A_RPE_post | A_RPE_pre | 0.670 |
| A_RPE_post | A_BPsys_post | 0.854 |
| A_RPE_post | A_HR_post | 0.691 |
| A_RPE_post | A_lactate_post | 0.787 |
| A_RPE_post | t_post | 0.709 |
| A_RPE_post | t_pre | 0.801 |
| A_RPE_post | S_post | 0.692 |
| A_RPE_post | S_pre | 0.801 |
| A_RPE_post | pW_R_post | 0.786 |
| A_RPE_pre | A_BPsys_pre | 0.712 |
| A_RPE_pre | t_pre | 0.781 |
| A_RPE_pre | nW_L_post | 0.670 |
| A_RPE_pre | nW_R_post | 0.677 |
| A_RPE_pre | S_pre | 0.781 |
| A_RPE_pre | pW_R_post | 0.695 |
| A_BPdia_post | A_BPsys_post | 0.764 |
| A_BPdia_pre | A_BPsys_post | 0.680 |
| A_BPsys_post | A_glucose_pre | 0.725 |

| | | |
|---|---|---|
| A_BPsys_post | A_BPsys_pre | 0.752 |
| A_BPsys_post | t_post | 0.673 |
| A_BPsys_post | t_pre | 0.677 |
| A_BPsys_post | S_pre | 0.677 |
| A_BPsys_post | DO2_gas_post | 0.793 |
| A_BPsys_post | DO2_vas_post | 0.684 |
| A_BPsys_post | pW_R_post | 0.804 |
| A_BPsys_post | Thb_gas_post | 0.709 |
| A_BPsys_post | Thb_gas_pre | 0.734 |
| A_BPsys_post | Thb_vas_pre | 0.724 |
| A_BPsys_pre | DO2_gas_pre | 0.709 |
| A_BPsys_pre | DO2_vas_post | 0.703 |
| A_BPsys_pre | Thb_vas_pre | 0.679 |
| A_glucose_post | A_lactate_post | 0.705 |
| A_glucose_post | t_post | 0.775 |
| A_glucose_pre | A_BPsys_pre | 0.816 |
| A_glucose_pre | A_HR_post | 0.810 |
| A_glucose_pre | A_lactate_post | 0.825 |
| A_glucose_pre | t_post | 0.815 |

| | | |
|---|---|---|
| A_glucose_pre | t_pre | 0.826 |
| A_glucose_pre | nW_L_post | 0.859 |
| A_glucose_pre | nW_R_post | 0.880 |
| A_glucose_pre | S_post | 0.798 |
| A_glucose_pre | S_pre | 0.826 |
| A_glucose_pre | pW_L_post | 0.734 |
| A_glucose_pre | pW_R_post | 0.727 |
| A_glucose_pre | Thb_gas_pre | 0.806 |
| A_glucose_pre | Thb_vas_post | 0.743 |
| A_glucose_pre | Thb_vas_pre | 0.761 |
| A_HR_post | A_BPsys_post | 0.730 |
| A_HR_post | A_lactate_post | 0.853 |
| A_HR_post | t_post | 0.929 |
| A_HR_post | t_pre | 0.787 |
| A_HR_post | S_pre | 0.787 |
| A_HR_post | Thb_gas_post | 0.672 |
| A_HR_post | Thb_vas_pre | 0.703 |
| A_HR_pre_rect | A_BPsys_pre | 0.738 |
| A_HR_pre_rect | A_lactate_pre | 0.886 |

| | | |
|---|---|---|
| A_HR_pre_rect | t_pre | 0.786 |
| A_HR_pre_rect | nW_L_post | 0.738 |
| A_HR_pre_rect | nW_R_post | 0.751 |
| A_HR_pre_rect | S_pre | 0.786 |
| A_HR_pre_rect | pW_R_post | 0.670 |
| A_HR_pre_rect | Thb_gas_pre | 0.848 |
| A_HR_pre_rect | Thb_vas_post | 0.760 |
| A_lactate_post | t_post | 0.893 |
| A_lactate_post | S_post | 0.931 |
| A_lactate_post | S_pre | 0.910 |
| A_lactate_post | pW_L_post | 0.853 |
| A_lactate_post | pW_R_post | 0.836 |
| A_lactate_post | Thb_gas_pre | 0.810 |
| A_lactate_post | Thb_vas_post | 0.781 |
| A_lactate_post | Thb_vas_pre | 0.820 |
| A_lactate_pre | pW_L_pre | 0.685 |
| t_post | nW_R_post | 0.949 |
| t_post | S_pre | 0.811 |
| t_post | pW_L_post | 0.855 |

| | | |
|---|---|---|
| t_post | pW_R_post | 0.829 |
| t_post | Thb_gas_post | 0.776 |
| t_post | Thb_gas_pre | 0.764 |
| t_post | Thb_vas_post | 0.944 |
| t_post | Thb_vas_pre | 0.746 |
| t_pre | A_lactate_post | 0.910 |
| t_pre | t_post | 0.811 |
| t_pre | nW_L_post | 0.874 |
| t_pre | nW_L_pre | 0.819 |
| t_pre | nW_R_post | 0.881 |
| t_pre | nW_R_pre | 0.804 |
| t_pre | S_post | 0.881 |
| t_pre | S_pre | 1.000 |
| t_pre | pW_L_post | 0.876 |
| t_pre | pW_L_pre | 0.769 |
| t_pre | pW_R_post | 0.880 |
| t_pre | pW_R_pre | 0.780 |
| t_pre | Thb_vas_post | 0.806 |
| nW_L_post | A_RPE_post | 0.750 |

| | | |
|---|---|---|
| nW_L_post | A_BPsys_post | 0.740 |
| nW_L_post | A_HR_post | 0.967 |
| nW_L_post | A_lactate_post | 0.891 |
| nW_L_post | t_post | 0.960 |
| nW_L_post | nW_L_pre | 0.984 |
| nW_L_post | nW_R_post | 0.996 |
| nW_L_post | nW_R_pre | 0.977 |
| nW_L_post | pW_L_post | 0.913 |
| nW_L_post | pW_R_post | 0.904 |
| nW_L_post | Thb_gas_post | 0.738 |
| nW_L_post | Thb_vas_post | 0.934 |
| nW_L_pre | A_glucose_pre | 0.794 |
| nW_L_pre | A_RPE_post | 0.721 |
| nW_L_pre | A_BPsys_post | 0.669 |
| nW_L_pre | A_HR_post | 0.961 |
| nW_L_pre | A_lactate_post | 0.864 |
| nW_L_pre | t_post | 0.976 |
| nW_L_pre | t_pre | 0.819 |
| nW_L_pre | nW_R_post | 0.968 |

| | | |
|---|---|---|
| nW_L_pre | nW_R_pre | 0.999 |
| nW_L_pre | S_post | 0.924 |
| nW_L_pre | pW_L_post | 0.865 |
| nW_L_pre | pW_R_post | 0.850 |
| nW_L_pre | Thb_gas_post | 0.763 |
| nW_L_pre | Thb_gas_pre | 0.776 |
| nW_L_pre | Thb_vas_post | 0.939 |
| nW_L_pre | Thb_vas_pre | 0.751 |
| nW_R_post | A_RPE_post | 0.731 |
| nW_R_post | A_BPsys_post | 0.745 |
| nW_R_post | A_HR_post | 0.964 |
| nW_R_post | A_lactate_post | 0.900 |
| nW_R_post | pW_L_post | 0.922 |
| nW_R_post | pW_R_post | 0.913 |
| nW_R_post | Thb_gas_post | 0.703 |
| nW_R_post | Thb_vas_post | 0.921 |
| nW_R_pre | A_glucose_pre | 0.779 |
| nW_R_pre | A_RPE_post | 0.731 |
| nW_R_pre | A_BPsys_post | 0.673 |

| | | |
|---|---|---|
| nW_R_pre | A_HR_post | 0.960 |
| nW_R_pre | A_lactate_post | 0.857 |
| nW_R_pre | t_post | 0.975 |
| nW_R_pre | nW_R_post | 0.958 |
| nW_R_pre | S_post | 0.912 |
| nW_R_pre | pW_L_post | 0.856 |
| nW_R_pre | pW_R_post | 0.840 |
| nW_R_pre | Thb_gas_post | 0.770 |
| nW_R_pre | Thb_gas_pre | 0.759 |
| nW_R_pre | Thb_vas_post | 0.930 |
| nW_R_pre | Thb_vas_pre | 0.735 |
| S_post | A_HR_post | 0.943 |
| S_post | t_post | 0.907 |
| S_post | nW_L_post | 0.954 |
| S_post | nW_R_post | 0.966 |
| S_post | S_pre | 0.881 |
| S_post | pW_L_post | 0.940 |
| S_post | pW_R_post | 0.926 |
| S_post | Thb_gas_pre | 0.833 |

| | | |
|---|---|---|
| S_post | Thb_vas_post | 0.862 |
| S_pre | nW_L_post | 0.874 |
| S_pre | nW_R_post | 0.881 |
| S_pre | nW_R_pre | 0.804 |
| S_pre | pW_L_pre | 0.769 |
| S_pre | pW_R_pre | 0.780 |
| S_pre | Thb_gas_pre | 0.966 |
| S_pre | Thb_vas_post | 0.806 |
| S_pre | Thb_vas_pre | 0.966 |
| DO2_gas_post | DO2_ave_gas_post | 0.917 |
| DO2_gas_post | DO2_vas_post | 0.824 |
| DO2_gas_post | Thb_gas_post | 0.752 |
| DO2_gas_pre | DO2_ave_gas_pre | 0.931 |
| DO2_gas_pre | DO2_ave_vas_pre | 0.817 |
| DO2_gas_pre | DO2_vas_post | 0.795 |
| DO2_gas_pre | DO2_vas_pre | 0.905 |
| DO2_vas_post | DO2_ave_vas_post | 0.841 |
| DO2_vas_post | DO2_vas_pre | 0.718 |
| DO2_vas_post | Thb_gas_post | 0.771 |

| | | |
|---|---|---|
| DO2_vas_pre | DO2_ave_vas_post | 0.847 |
| DO2_vas_pre | DO2_ave_vas_pre | 0.929 |
| DO2_vas_pre | ave_tHb_vas_pre | 0.803 |
| DO2_vas_pre | A_BPsys_pre | 0.682 |
| sP_week1_2 | sP_week3_4 | 0.976 |
| sP_week1_2 | sP_week5_6 | 0.956 |
| sP_week1_2 | sP_week7_8 | 0.919 |
| sP_week3_4 | sP_week5_6 | 0.996 |
| sP_week3_4 | sP_week7_8 | 0.973 |
| sP_week3_4 | Thb_gas_post | 0.669 |
| sP_week5_6 | sP_week7_8 | 0.984 |
| sP_week5_6 | Thb_gas_post | 0.689 |
| sP_week7_8 | Thb_gas_post | 0.678 |
| sP_week7_8 | Thb_vas_post | 0.675 |
| P_week1_2 | ave_tHb_gas_pre | 0.814 |
| P_week1_2 | P_week3_4 | 0.993 |
| P_week1_2 | P_week5_6 | 0.975 |
| P_week1_2 | P_week7_8 | 0.957 |
| P_week3_4 | P_week5_6 | 0.991 |

| | | |
|---|---|---|
| P_week3_4 | P_week7_8 | 0.978 |
| P_week5_6 | ave_tHb_vas_post | 0.711 |
| P_week5_6 | P_week7_8 | 0.997 |
| P_week7_8 | ave_tHb_gas_pre | 0.868 |
| P_week7_8 | ave_tHb_vas_post | 0.735 |
| P_week7_8 | pW_L_pre | 0.684 |
| pW_L_post | A_RPE_post | 0.765 |
| pW_L_post | A_BPsys_post | 0.784 |
| pW_L_post | A_HR_post | 0.900 |
| pW_L_post | S_pre | 0.876 |
| pW_L_post | pW_L_pre | 0.715 |
| pW_L_post | pW_R_post | 0.997 |
| pW_L_post | pW_R_pre | 0.719 |
| pW_L_post | Thb_gas_post | 0.689 |
| pW_L_post | Thb_gas_pre | 0.899 |
| pW_L_post | Thb_vas_post | 0.861 |
| pW_L_pre | A_RPE_pre | 0.848 |
| pW_L_pre | pW_R_post | 0.741 |
| pW_L_pre | pW_R_pre | 0.994 |

| | | |
|---|---|---|
| pW_L_pre | Thb_gas_pre | 0.877 |
| pW_L_pre | Thb_vas_post | 0.679 |
| pW_R_post | A_HR_post | 0.886 |
| pW_R_post | S_pre | 0.880 |
| pW_R_post | pW_R_pre | 0.744 |
| pW_R_post | Thb_gas_post | 0.685 |
| pW_R_post | Thb_gas_pre | 0.910 |
| pW_R_post | Thb_vas_post | 0.844 |
| pW_R_post | Thb_vas_pre | 0.884 |
| pW_R_pre | A_RPE_pre | 0.862 |
| pW_R_pre | A_lactate_pre | 0.704 |
| pW_R_pre | sP_week5_6 | 0.698 |
| pW_R_pre | sP_week7_8 | 0.693 |
| pW_R_pre | Thb_gas_pre | 0.885 |
| pW_R_pre | Thb_vas_post | 0.714 |
| pW_R_pre | Thb_vas_pre | 0.889 |
| Thb_gas_post | Thb_vas_post | 0.846 |
| Thb_vas_post | A_HR_post | 0.856 |
| Thb_vas_pre | A_RPE_post | 0.815 |

| | | |
|---|---|---|
| Thb_vas_pre | A_RPE_pre | 0.843 |
| Thb_vas_pre | t_pre | 0.966 |
| Thb_vas_pre | nW_L_post | 0.822 |
| Thb_vas_pre | nW_R_post | 0.829 |
| Thb_vas_pre | S_post | 0.807 |
| Thb_vas_pre | pW_L_post | 0.870 |
| Thb_vas_pre | pW_L_pre | 0.884 |
| Thb_vas_pre | Thb_vas_post | 0.805 |

**Supplemental table 4:** *Retrospective power analysis.* List summarizing the calculated effect size f and power for the applied statistical model of a repeated ANOVA for the within factor `training` (T) and the between factor `protocol` as calculated using G*Power for the statistical sizes (alpha p-values, effect size ◎2 and the correlation of repeated measures) for within-between interactions for repeated-measures ANOVA that revealed from the descriptive analysis of the results with SPSS.

| **parameter type** | **parameter** | **exercise type** | **exercise device** | **alpha (P) between** | **alpha (T) within** | **alpha (T x P) within x between** | **h2 (TxP)** | **r-value** | **effect size f within-between interactions** | **Power (1-beta)** |
|---|---|---|---|---|---|---|---|---|---|---|
| muscle strength | PPP | interval type exercise | soft robot | 0.829 | <0.001 | 0.043 | 0.467 | 0.970 | 0.936 | 0.999 |
| muscle strength | NPP | interval type exercise | soft robot | 0.591 | 0.022 | 0.391 | 0.107 | 0.580 | 0.346 | 0.498 |
| cardiopulmonary | RPE | interval type exercise | soft robot | 0.004 | 0.004 | 0.015 | 0.072 | 0.810 | 0.279 | 0.962 |
| cardiovascular | serum glucose concentration 0.558 | | interval type exercise | | soft robot | <0.001 | 0.638 | 0.031 | 0.058 | 0.500 0.248 |
| muscle metabolism | blood lactate concentration | interval type exercise | soft robot | 0.001 | 0.001 | 0.050 | 0.050 | 0.870 | 0.230 | 0.997 |
| cardiopulmonary | systolic blood pressure | interval type exercise | soft robot | 0.012 | 0.405 | 0.001 | 0.131 | 0.880 | 0.388 | 0.998 |
| cardiopulmonary | diastolic blood pressure | interval type exercise | soft robot | <0.001 | 0.500 | 0.200 | 0.021 | 0.690 | 0.147 | 0.800 |
| cardiopulmonary | heart rate | interval type exercise | soft robot | 0.009 | 0.092 | 0.009 | 0.092 | 0.930 | 0.318 | 0.999 |
| muscle metabolism | total O2 deficit | interval type exercise | soft robot | 0.096 | 0.006 | 0.037 | 0.274 | 0.700 | 0.614 | 0.815 |
| muscle metabolism | average O2 deficit | interval type exercise | soft robot | 0.782 | 0.473 | 0.036 | 0.277 | 0.880 | 0.619 | 1.000 |
| muscle metabolism | total tHb | interval type exercise | soft robot | <0.001 | <0.001 | 0.092 | 0.189 | 0.290 | 0.483 | 0.908 |
| muscle metabolism | average tHb | interval type exercise | soft robot | 0.365 | 0.002 | 0.236 | 0.099 | 0.370 | 0.332 | 0.617 |
| cardiopulmonary | RPE | ramp exercise | cycle ergometer | <0.001 | 0.011 | 0.261 | 0.029 | 0.810 | 0.173 | 0.519 |
| muscle metabolism | lactate | ramp exercise | cycle ergometer | <0.001 | 0.100 | <0.001 | 0.296 | 0.870 | 0.648 | 1.000 |

| | | | | | | | | | | |
|---|---|---|---|---|---|---|---|---|---|---|
| cardiovascular | glucose | ramp exercise | cycle ergometer | 0.572 | 0.273 | 0.497 | 0.011 | 0.500 | 0.106 | 0.100 |
| cardiopulmonary | heart rate | ramp exercise | cycle ergometer | 0.045 | 0.631 | 0.007 | 0.153 | 0.930 | 0.425 | 1.000 |
| cardiopulmonary | systolic blood pressure | ramp exercise | cycle ergometer | <0.001 | 0.628 | 0.148 | 0.047 | 0.880 | 0.222 | 0.940 |
| cardiopulmonary | diastolic blood pressure | ramp exercise | cycle ergometer | 0.854 | 0.007 | 0.186 | 0.039 | 0.690 | 0.202 | 0.477 |
